# Potential Invasive Indo‐Pacific *Acropora* in a Coral Reef of Venezuela: A Contribution to Their Morphological and Molecular Knowledge

**DOI:** 10.1002/ece3.73167

**Published:** 2026-03-17

**Authors:** Estrella Y. Villamizar G., Rossana C. Jaspe, Anaurora Yranzo‐Duque, Jeannette Pérez‐Benítez, Yoneira F. Sulbarán, Samuel Narciso, Bryan A. Rujano‐Espinoza, Carlos Pereira, María C. Goite, Flor H. Pujol

**Affiliations:** ^1^ Laboratorio de Ecología de Sistemas Acuáticos, Centro de Ecología y Evolución, Instituto de Zoología y Ecología Tropical Universidad Central de Venezuela Caracas Venezuela; ^2^ Laboratorio de Virología Molecular, Centro de Microbiología y Biología Celular Instituto Venezolano de Investigaciones Científicas Altos de Pipe Venezuela; ^3^ Fundación Para la Defensa de la Naturaleza (FUDENA) Caracas Venezuela; ^4^ Laboratorio de Biología Marino Costera (BioMaC‐UC), Departamento de Biología Universidad de Carabobo Valencia Venezuela; ^5^ Laboratorio de Taxonomía y Ecología de Macrofitas Marinas, Centro de Botánica Tropical, Instituto de Biología Experimental Universidad Central de Venezuela Caracas Venezuela; ^6^ Laboratorio de Química de Metales de Transición, Centro de Química, Instituto Venezolano de Investigaciones Científicas IVIC Altos de Pipe Venezuela

**Keywords:** *Acropora tenuis* group, Early detection rapid response (EDRR), Indo‐Pacific, invasive species, molecular analysis, morphological characterization, Venezuela‐southern Caribbean

## Abstract

The introduction of invasive species into marine environments is a global crisis, driving significant biodiversity loss. Here, we report the first record of a non‐native hermatypic coral in Venezuela, exhibiting biological traits characteristic of invasive species. The coral, a member of the genus *Acropora* (Scleractinia: Acroporidae) native to the Indo‐Pacific, was occupying a discrete 11 m^2^ patch within a reef in Morrocoy National Park, a Marine Protected Area (MPA). Due to the high ecological risk, and with authorization from environmental authorities, we removed all colonies while representative samples were preserved for morphological and molecular characterization. To monitor for recurrence—either from residual fragments or secondary introductions—surveys were conducted every four to 6 months between 2023 and 2025. This intervention follows Early Detection and Rapid Response (EDRR) protocols, emphasizing the necessity of continued surveillance at affected sites. Phylogenetic analysis assigned the non‐native specimens to the *Acropora tenuis* (Dana, 1846) group. The location and species suggest a probable introduction via the marine aquarium trade. Stringent enforcement of environmental regulations and rigorous monitoring of the aquarium trade are imperative to prevent further introductions and the consequent and irreversible loss of marine biodiversity and ecosystem services.

## Graphical abstract

We report the first record of a non‐native hermatypic coral in Venezuela, exhibiting biological traits characteristic of invasive species. The coral is a member of the genus Acropora (Scleractinia: Acroporidae), native to the Indo‐Pacific. This non‐native petrous coral occurred in a reef of the Morrocoy National Park, in the southern Caribbean. We removed all non‐native colonies. This intervention follows Early Detection and Rapid Response (EDRR) protocols, emphasizing the necessity of continued surveillance at affected sites

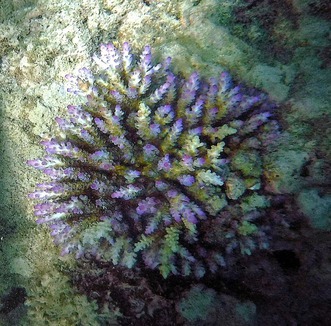

## 1 Introduction

The progressive degradation and loss of live coral cover reefs worldwide are well‐documented and concerning phenomena (Hughes et al. 2017, 2018, Souter et al. 2021, De'ath et al. 2012). This decline is particularly critical in Caribbean coral reefs, which have experienced massive coral loss spanning more than four decades (Souter et al. 2020). Several anthropogenic drivers have been identified as major causes of this degradation, including overfishing, coastal pollution, global warming, and the introduction of invasive non‐native species (Jackson et al. 2014; Hughes et al. 2018; Cramer et al. 2021; Gutierrez et al. 2024).

Biological invasions are now recognized as the second leading cause of biodiversity loss in both terrestrial and aquatic systems worldwide (IPBES 2019; IUCN 2000; Mack et al. 2000; Mayfield et al. 2021). The rate of non‐native species introduction has accelerated markedly in recent decades. For instance, Bailey et al. (2020) reported that, over a 50 year period (1965–2015), a new invasive species was detected every 8.4 days across 49 global ecosystems, spanning marine, estuarine, and freshwater environments such as the Laurentian Great Lakes. Increasing globalization has enabled invasive species to bypass natural geographic and oceanographic barriers, via diverse transport pathways, including commercial shipping, aviation and offshore infrastructure (semi‐submersible platforms, oil, and other full tankers). Key anthropogenic vectors facilitating this invasions include the construction of artificial canals, aquaculture, ballast water discharge, biofouling on mobile floating platforms, marine litter, and the aquarium trade (Katsanevakis et al. 2013; Galil and Gevili 2014; Creed et al. 2016; Bailey et al. 2020).

The aquarium trade is a long‐standing global industry centered on the international commerce of marine ornamental species (Bruckner 2005). The risk of introducing non‐native species through this activity is not limited to the intentionally transported organisms but extends to associated symbionts and hitchhiking taxa co‐introduced with the target species (Patoka et al. 2020). This trade encompasses a diverse array of taxa, including fishes, scleractinian corals, and various invertebrates such as octocorals, anemones, crustaceans, and echinoderms (Bruckner 2005; Palmtag 2017; Pavitt et al. 2021).

Global demand for marine biota, particularly cnidarians, has increased in recent decades (Rhyne et al. 2017; Akmal et al. 2020). Between 1990 and 2016, 97% of all recorded export transactions of marine animals worldwide (excluding mammals, birds, and reptiles) involved Anthozoans and Hydrozoans, with the volume of transactions exhibiting a sevenfold increase between 2007 and 2016 (Pavitt et al. 2021). Corals were almost entirely exported as wild‐sourced live and raw material (coral rock, live rock, and substrate), totaling 19.8 million live coral pieces and 24 million kg of raw coral, predominantly scleractinian species (Pavitt et al. 2021). The Asian Pacific region—particularly Australia, Fiji, and Indonesia—constitutes the primary exporting hub, whereas the United States remains the predominant global importer (Sosnowski et al. 2020; Pavitt et al. 2021).

Although numerous exotic and invasive marine invertebrate species have been reported in Venezuela, formal peer‐reviewed records are limited (Pérez et al. 2007; Figueroa López and Brante 2020), and much of the existing information is confined to gray literature and unpublished reports, resulting in a significant knowledge gap within the indexed scientific record. Documented taxa include crustaceans, mollusks, ascidians, annelids, and cnidarians, with crustaceans being the most numerous group (Pérez et al. 2007; Figueroa López and Brante 2020; González 2022). To date, five non‐native cnidarians have been reported in Venezuela—two hydrozoans and three anthozoans (Urich 1977; Capelo et al. 2004; Govindarajan et al. 2005; Ruiz Allais et al. 2014; Hernández et al. 2018; Fofonoff et al. 2020). On the basis of a synthesis of 80 marine non‐native species, ballast water remains the primary introduction vector for the Venezuelan coast. Among these, the octocoral *Unomia stolonifera* has emerged as a critical threat to coastal ecosystems, following its illegal introduction and deliberate seeding by commercial aquarists between 2000 and 2007, in a hard substrate in northeastern Venezuela (Ruiz Allais et al. 2014, 2021; Benayahu et al. 2021). In this study, we report the first record of a non‐native hermatypic coral in Venezuela, and describe the rapid‐response measures implemented to prevent its spread. This species, belonging to the *Acropora* genus, was discovered within Morrocoy National Park (MNP). We provide a general morphological description, and its molecular identity inferred from mitochondrial DNA barcoding.

## 2 Materials and Methods

### 2.1 Study Area and Sampling Design

**FIGURE 1:**
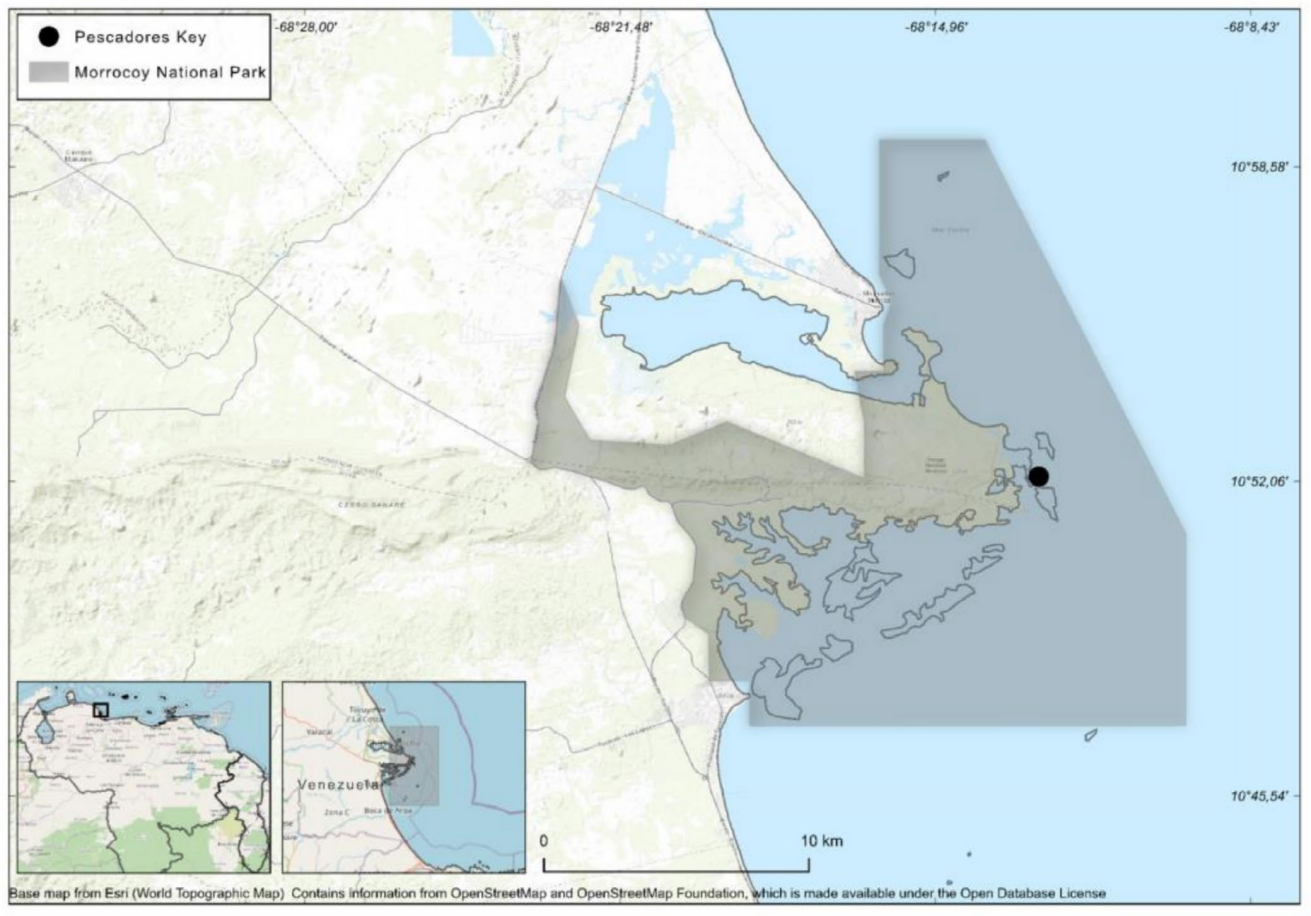
Geographic location of the studied site. Map of Venezuela (North of South America, bottom left) with a square indicating the region of “Golfo Triste” (bottom right) and an enlarged map of the studied area indicating the boundaries of Morrocoy National Park (shaded area) and the precise location where the non‐native *Acropora* was found (black dot).

The non‐native coral was initially detected in march 2019 within the back‐reef zone of Pescadores Key, located on the northwestern continental coast of Venezuela (10°52.9′32″N and 68°12.50′13″W, Figure 1), at depths of 1–2 m. Due to logistical and funding constraints, subsequent monitoring was deferred until late 2022, at which point we identified two large clusters (comprising approximately 10 and 20 colonies, respectively), one small group of four colonies, and a single isolated colony. Several colonies exhibited signs of compromised health. The site was subsequently monitored every 4–6 months from December 2022 to September 2024, with a final survey conducted in May 2025.

All colonies observed at Pescadores Key were removed in April 2023 under ministerial authorization (Permit Nos. 059060068). Removal was performed by manually detaching colonies from the substrate with a knife (stainless‐steel blades). Since the species is easily fractured, the attachment sites were exhaustively inspected to eliminate residual fragments and prevent asexual propagation. Despite these efforts, a small number of new colonies were detected and removed during subsequent surveys. (September and December 2023, April and September 2024, and May 2025). Representative specimens were preserved for laboratory morphological examination and molecular analysis.

### 2.2 Benthic Characterization

**FIGURE 2:**
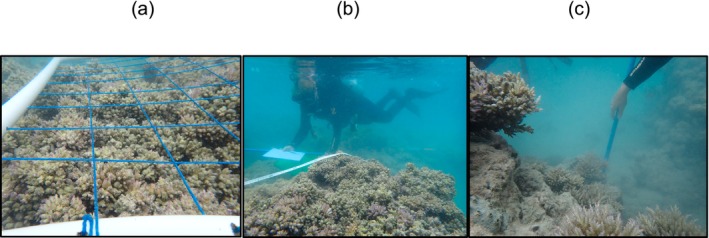
Field survey equipment and procedures. (a–b) quadrat 1 m^2^ for coverage estimation of non‐native corals and other benthic categories. (c) Calibrated rod for morphometric measurements of coral colonies.

Prior to remotion, we estimated the live cover of both healthy and unhealthy *Acropora* sp. colonies, and the size of distinguishable colonies. For distinct colonies, maximum colony diameter and height (from the base to the highest apical branch) were measured. Additionally, the coverage of associated benthic categories—including hydrocorals, sand, and coral rubble—was estimated using 1 m square quadrat (Figure 2).

We identified the species by comparing the morphological characters of the specimen to published taxonomic guides (Wallace and Dai 1997, C. C. Wallace 1999, Wallace et al. 2012, Smithsonian National Museum of Natural History online database, n.d.), and with molecular analysis. However, due to discrepancies between traditional taxonomy and modern systematics found in the literature, the final identification followed the most recent taxonomic revision by Bridge et al. (2024), which integrates both morphological and molecular data.

The morphological description included colony coloration, branch shape, the arrangement of axial and radial corallites, and the coenosteum appearance. Additionally, we recorded branch length and width, as well as the internal and external diameters of the axial corallites.

Following remotion, the total biomass of the collected Acropora fragments was determined using a weight with a precision of 1 kg (maximum capacity 25 kg). Subsequently, all fragments were carefully inspected to collect associated macrofauna for taxonomic identification in the laboratory.

### 2.3 Molecular Analysis

#### 2.3.1 DNA Extraction, Amplification, and Sequencing

We preserved tissue samples in 99% ethanol and stored at −20°C. Total DNA was extracted using the DNeasy Blood and Tissue Kit from Qiagen (Hilden, Germany) following the manufacturer's protocol. Extracted DNA was stored at −80°C. To amplify the internal transcribed spacer (ITS) of the nuclear ribosomal RNA gene, we used the primers 1S (5′‐GGT ACC CTT TGT ACA CAC CGC CCG TCG CT‐3′) and 2SS (5′‐GCT TTG GGC GGC AGT CCC AAG CAA CCC GAC TC‐3′) as described by Wei et al. (2006). The PCR reaction contained 5 μL of DNA, 1 μL of 10 mM dNTP Mixture, 5 μL of 10X PCR Buffer—Mg, 2.5 μL of 50 mM MgCl**_**2, 1 μL of each 10 mM primer, 0.5 μL of Platinum Taq DNA Polymerase (Thermo Fisher Scientific), and 34 μL of sterile distilled water until a final volume of 50 μL. Amplification was performed (ESCO Swift Max Pro Thermal Cycler) with the following thermal profile: 120 s at 94°C, 40 cycles of 30 s at 94°C, 30 s at 58°C, 60 s at 72°C with a final extension of 5 min at 72°C. Amplified fragments were checked on 1% agarose gel electrophoresis. A variable region of the Mitochondrial gene (1255 nt) was amplified and sequenced, spanning the mitochondrial gene from nt 11,462 to 12,717 (according to the accession number of the coral isolate AF338425), with primers designed to cover this region, Ps (5′‐TCAGTGTTAGTACAAATTGCCCG‐3′) and Pr (5′‐GCTCAACCAAATGATATGGATGAC‐3′). The PCR reaction was the same to that used for the ITS region, with the following cycling conditions: 94°C for 2 min, followed by 35 cycles at 94°C for 30 s, 55°C for 60 s, and 72°C for 120 s, and a final extension of 72°C for 5 min. PCR fragments were sent to Macrogen Sequencing Service (Macrogen, Korea) for sequencing.

### 2.4 Phylogenetic Analysis

The evolutionary history was inferred by using the Maximum Likelihood method and the Hasegawa‐Kishino‐Yano model (Hasegawa et al. 1985). The tree with the highest log likelihood (−2484.75) is shown. The percentage of trees in which the associated taxa clustered together (500 replicates) is shown below the branches. Initial tree(s) for the heuristic search were automatically generating by applying Neighbor‐Join and BioNJ algorithms to a matrix of pairwise distances estimated via the Maximum Composite Likelihood (MCL) approach, followed by selecting the topology with the superior log‐likelihood value. Evolutionary rate differences among sites were modeled using a discrete Gamma distribution (5 categories, +G, parameter = 0.0500). The tree was drawn to scale, with branch lengths representing the number of substitutions per site. The final dataset included a total of 1344 positions. Codon positions included were 1st + 2nd + 3rd + Noncoding. All phylogenetic analyses were performed in MEGA11 with a bootstrap value of 500 (Tamura et al. 2021).

## 3 Results

### 3.1 Morphological Description of the Colonies

**FIGURE A1:**
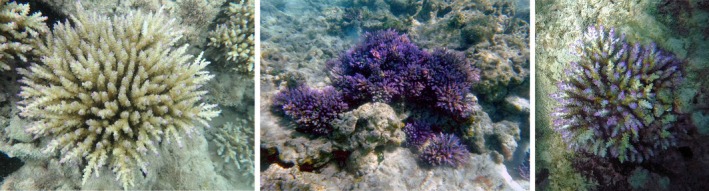
Colonies of the non‐native *Acropora tenuis* species group, showing differences in colony size and coloration patterns.

**FIGURE A2:**
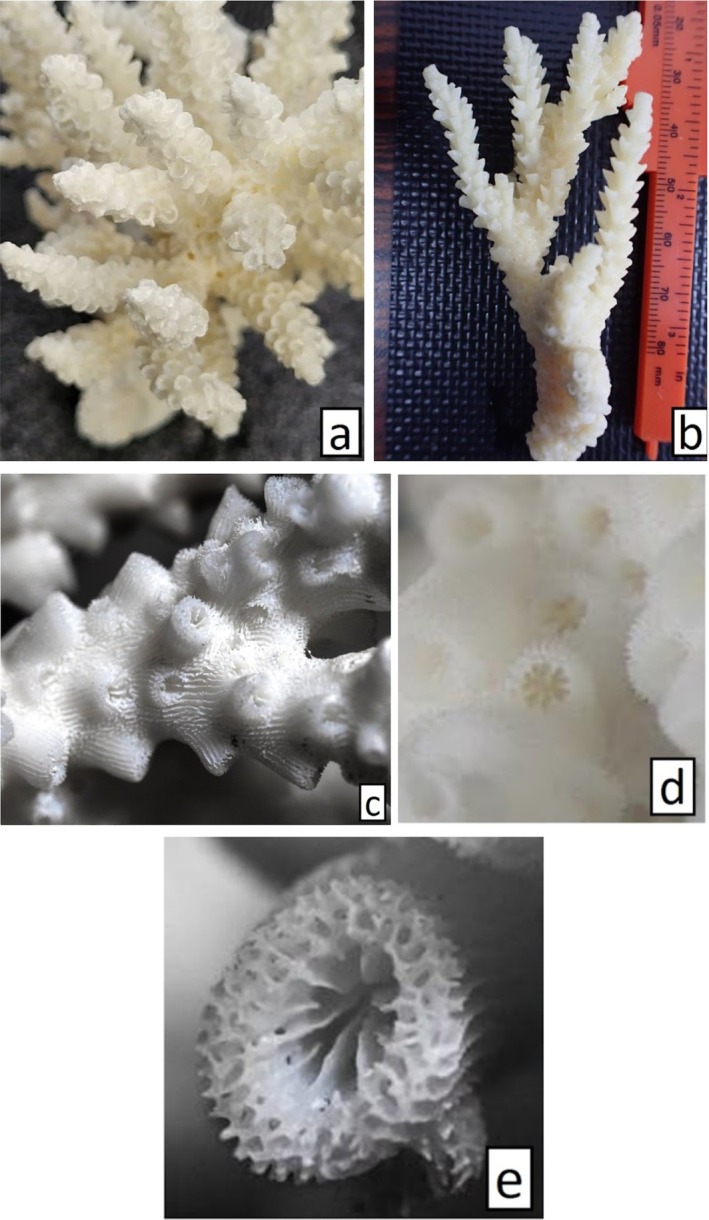
General colony view showing radial corallite arrangement (a), Branch detail showing axial and radial corallites (b). Lateral view of a branch showing coenosteum on and between radial corallites (c). Close view showing primary and secondary septa in radial corallites (d). (e) Detail of primary and secondary septa in a radial corallite (apical view).

Colonies are corymbose (Appendix 1: Figure A1). Branches are elongated and slender, with a maximum length of 12.81 cm, and a maximum thickness of 1.15 cm (Table 1). Axial corallites are tubular with rounded margins. Radial corallites are densely arranged in certain sections of the colony (Appendix 2: Figure A2D), their morphology is variable but predominantly cochleariform (Appendix 2: Figure A2A), showing subtle gradations in length and shape (from appressed tubular to cochleariform) from the branch tips to the base (Table 1, Appendix 2: Figure A2B,C). The coenosteum is costate with simple spinules (Appendix 2: Figure A2C). Live coloration is typically cream with purple branch tips, although some colonies exhibit a uniform purple hue. In both axial and radial corallites, the septa do not reach the center of the calice (Appendix 2: Figure A2D–E). Primary septa extend to 2/3R, whereas secondary septa reach 1/4 R in radial corallites.

**TABLE 1:** Morphometric characterization of branches and axial corallites in non‐native *Acropora* (*n* = 19).

	Branches length (cm)	Branches max Ø (cm)	Axial corallite ext. Ø (mm)	Axial corallite int. Ø (mm)	Radial corallite ext. Ø (mm)	Radial corallite int. Ø (mm)
Average	9.73	0.75	2.42	1.17	1.65	0.82
Std. Err.	0.44	0.05	0.16	0.10	0.12	0.14
Maximum	12.81	1.15	4.50	2.30	2.00	1.40
Minimum	6.78	0.47	1.20	0.70	1.30	0.50

*Note:* Parameters include length, maximum diameter, and internal/external corallite diameters (Ø). Data Matrix in Appendix 7, Table A3.

Abbreviations: ext., external; int., internal; Max, maximum.

### 3.2 Ecological Description

**FIGURE A3:**
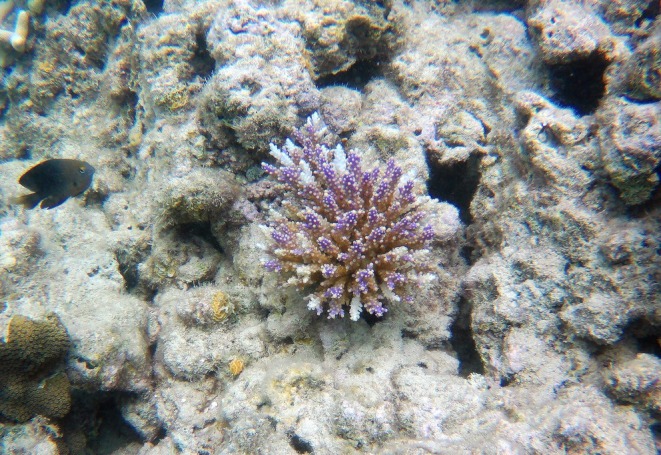
First colony of non‐native *Acropora* recorded at Pescadores Key, Morrocoy National Park, in March 2019. Photography: Rujano (2019).

In 2019, we found only one colony of *Acropora* in the MNP. The colony was growing over a dead coral colony colonized by turf algae (Appendix 3: Figure A3). This colony was relatively small, with a maximum diameter of 23.5 cm, and a mean height of 12 cm (Villamizar et al. 2022). The absence of additional colonies throughout the total surveyed area (26,136.3 m^2^) supports the hypothesis of an illegal, localized introduction. From 2019 to the end of 2022, the population of the non‐native *Acropora* continued to increase significantly, proliferating into two large clusters (minimum of 10 and 20 colonies, respectively), one small cluster (four colonies), and one isolated colony. These formed a dense, interlaced framework where individual boundaries were often indistinguishable. Within the invaded patch, the mean cover of healthy *Acropora* sp. was 13.72% (±3.29 SE), while unhealthy *Acropora* sp. cover was 68.18% (±9.97 SE). Other benthic categories included *Millepora alcicornis* (1% ± 0.81% SE), sand (10.54% ± 8.32% SE), and coral rubble over sand (6.54% ± 6.53% SE).

Due to high colony density and structural fusion, only 11 colonies could be measured in situ for full morphometrics (Table 2). Additionally, maximum longitudinal diameters for another 15 colonies were estimated via photography analysis (See Appendix 7, Table A4 (b)).

**TABLE 2:** Morphometric measurements (longitudinal diameter, transverse diameter, and height) of non‐native *Acropora* colonies (*n* = 11) measured in situ at Pescadores Reef in April 2023.

	Cross‐sectional max. diameter (cm)	Transv. diameter (cm)	Height (cm)
Average	18.64	15.00	16.36
Std. error	2.14	1.65	2.03
Max	35.00	25.00	25.00
Min	5.00	5.00	5.00

Abbreviations: Max., maximum; Min., minimum; Transv., transverse.

**FIGURE 3:**
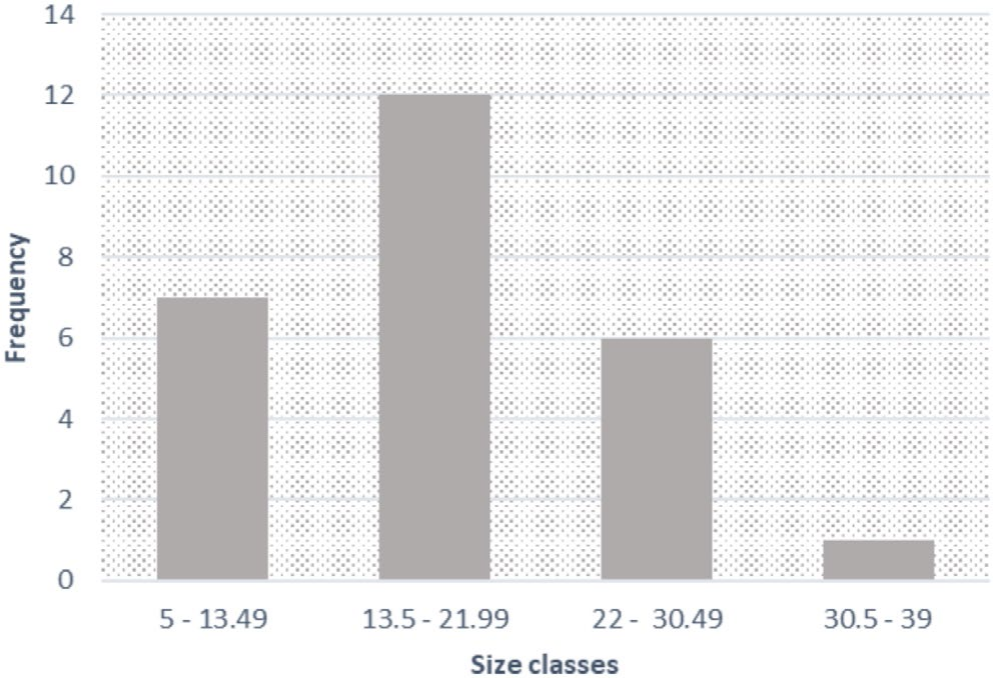
Population size structure of non‐native *Acropora* colonies (*n* = 26) at Pescadores Key, Morrocoy National Park (April 2023).

Considering the total number of colonies measured in situ and those measured with photographic analysis, it was possible to obtain an approximation of the population size‐frequency distribution of the non‐native *Acropora* (Figure 3).

The size–frequency distribution shows a predominance of colonies in the 13.5–21.99 cm range (likely pre‐adults). Similar frequencies were observed in the 5–13.5 cm (juvenile) and 22–30.5 cm (pre‐adult or adult) size classes, with only a single colony exceeding 30.5 cm (adult). On the basis of this distribution, the species poses a significant risk to native coral communities; a high percentage of individuals have reached or are nearing sexual maturity, potentially spawning within this Marine Protected Area (MPA). Consequently, the removal of these colonies was deemed necessary to mitigate further ecological damage to the reefs.

### 3.3 Macrofauna Associated With Non‐Native *Acropora* Colonies

A total of three phyla, four classes, fourteen families and nineteen invertebrate species (11 mollusks, 7 arthropods, and 1 echinoderm) were found in a volume of 20.84 L and a dry weight of 83 Kg. of the non‐native *Acropora* colonies extracted from the study area (Table 3). All the species found are native to the Caribbean.

**TABLE 3:** Taxonomic composition of macrofauna associated with non‐native *Acropora* colonies collected at Pescadores Reef in April 2023.

Phylum	Class	Family	Species
Mollusca	Gastropoda	Cerithiidae	*Cerithium litteratum* (Born, 1778)
		Muricidae	*Coralliophila galea* (Dillwyn, 1823)
		Vermetidae	*Petaloconchus erectus* (Dall, 1888)
			*Petaloconchus varians* (A. d'Orbigny, 1839)
			*Dendropoma corrodens* (A. d'Orbigny, 1841)
		Triviidae	*Pusula pediculu*s (Lineo, 1758)
		Pinanidae	*Engina turbinella* (Kiener, 1836)
		Pseudomelatomidae	*Crassispira nigrescens* (C.B. Adams, 1845)
	Bivalvia	Arcidae	*Barbatia cancellaria* (Lamarck, 1819)
			*Lamarcka imbricata* (Bruguière, 1789)
		Isognomonidae	*Isognomon radiatus* (Anton, 1838)
Arthropoda	Malacostraca	Mithracidae	*Mithraculus sculptus* (Lamarck, 1818)
			*Mithraculus* sp.
		Xanthidae	*Cataleptodius floridanus* (Gibbes, 1850)
		Diogenidae	*Paguristes* sp.
			*Calcinus tibicen* (Herbst, 1791)
		Gonodactylidae	*Neogonodactylus curacaoensis* (Schmitt, 1924)
		Alpheidae	*Synalpheus* sp.
Echinodermata	Ophiuroidea	Ophiodermatidae	*Ophioderma panamense* Lütken, 1859

### 3.4 Molecular Characterization and Phylogenetic Analysis

A Blast analysis of the 359 nt of the ITS sequence revealed > 99% identity with sequences from *Acropora tenuis* (GenBank accession numbers Nos. AF538488 and AF538489).

**FIGURE 4:**
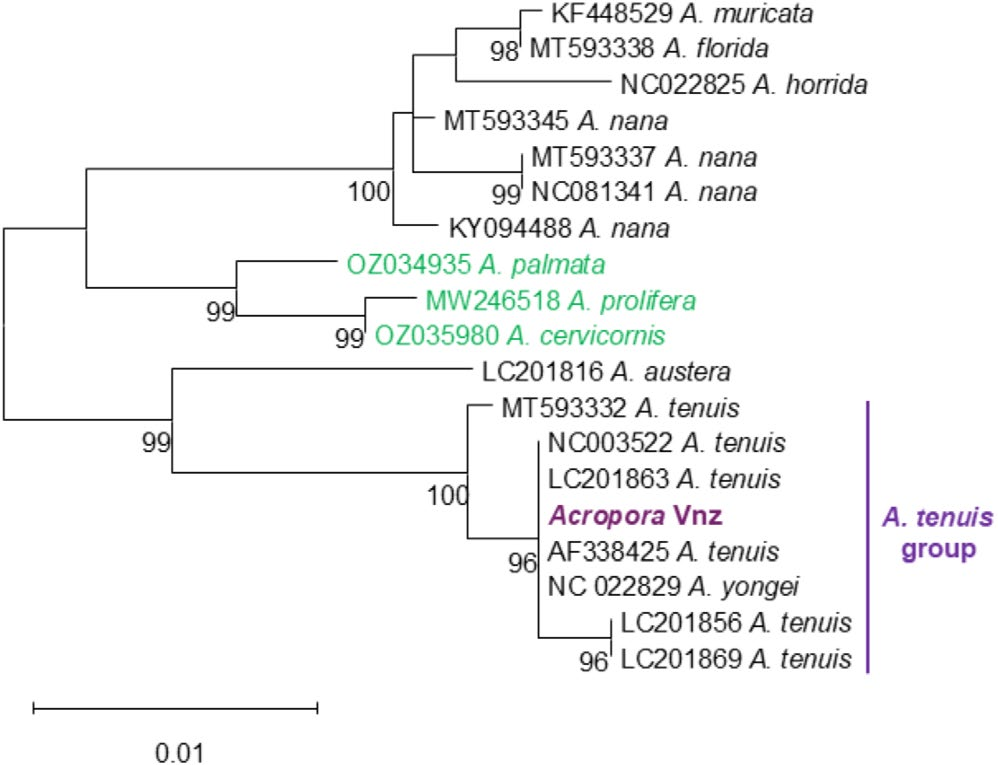
Maximum likelihood phylogenetic tree of the non‐native *Acropora* specimens. Labels for each taxon include the GenBank accession number followed by the species name. The sequence of the non‐native *Acropora* is highlighted in purple, while sequences of autochthonous *Acropora* species are indicated in green. Distant congeners were included as the outgroup. Bootstrap support values (500 replicates) are shown at the nodes.

**FIGURE A4:**
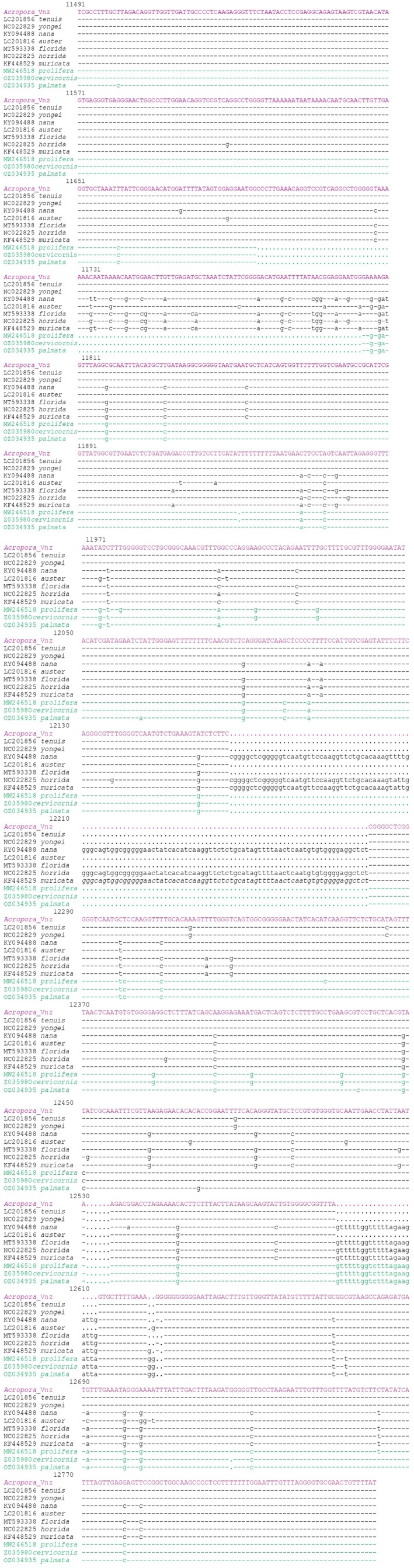
Sequence alignment of the mitochondrial gene. Sequences from non‐native *Acropora* in this study are highlighted in pink; native Caribbean *Acropora* sequences are shown in green.

To examine the phylogenetic placement of the Venezuelan specimens, we compared a partial region of the mitochondrial genome with reference sequences from NCBI GenBank. Although mitochondrial markers evolve slowly in corals and other metazoans (Huang et al. 2008) and is not capable of resolving species‐level relationships in *Acropora* (Ramírez‐Portilla et al. 2022), it is nonetheless useful for providing some insight into the identity of the species to support the morphological analysis (Appendix 4: Figure A4). The alignment of this genomic region clearly differentiated the major clades within the *Acropora* genus. Phylogenetic analysis confirmed the placement of the non‐native colonies within the *Acropora tenuis* (Dana 1846) species group, exhibiting 100% identity with several *A. tenuis* isolates. Notably, the sequence also matched with an *A. yongei* isolate, which is morphologically distinct from our non‐native species. In contrast, the Venezuelan sequence displayed significantly lower identity (96%–97%) with all other congeners included in the analysis (Figure 4).

## 4 Discussion

*Acropora* is the most diverse (> 100 species) and ecologically dominant coral taxon in the Indo‐Pacific (Wallace 2012; Wallace et al. 2012; Bridge et al. 2024). In contrast, only three extant species occur in the Atlantic: *A. palmata* , *A. cervicornis* and *A. prolifera* , which is considered a hybrid of the former two species (Vollmer and Palumbi 2002; Precht et al. 2019). At the time of the opportunistic encounter in 2019, the presence of a single, isolated *Acropora* colony in Morrocoy National Park was unexpected, as only small colonies of *A. palmata* had been previously recorded in the area. This individual was initially misidentified as *A. cervicornis* based solely on photographic evidence (Villamizar et al. 2022). However, no further taxonomic validation was carried out for the following 2 years. It was not until December 2022 that we visited the area, finding numerous colonies that clearly diverge from the known Caribbean species and aligned closely with the Indo‐Pacific morphotypes. The rapid transition from a single colony to a dense framework within 3 years highlights the invasive potential of this taxon in a non‐native environment.

Traditionally, coral taxonomy has been based on skeletal morphology; however, some characters are often highly variable (Veron 1995; Todd 2008). Sadek et al. (2018) studied the morphological variations of eight scleractinian corals inhabiting the Red Sea and the Arabian Gulf and found that in seven of the eight species, corallite diameter is highly influenced by geographic distribution. Furthermore, the processes of evolutionary convergence of morphological characters can further complicate the identification of species based solely on their morphology (Budd et al. 2010; Grupstra et al. 2024). Over the last three decades, molecular taxonomy and phylogenetics have been increasingly used to resolve coral systematics (Van Oppen et al. 2000, 2001; Richards et al. 2013; Bridge et al. 2024). These molecular tools are particularly useful for diverse genera such as *Acropora*, where high morphological similarity among certain species often leads to taxonomic ambiguity (Bridge et al. 2024). Consequently, identification of the non‐native *Acropora* in this study, based solely on macromorphological traits, was not achieved with complete certainty. However, considering the results of the phylogenetic analysis, it clearly places the non‐native *Acropora* within the *Acropora tenuis* species group (Clade I, Cowman et al. 2020), This molecular assignment is further supported by morphological comparisons, as the specimens share diagnostic characters with the species *Acropora tenuis* , as is shown in Appendix 5: Table A1.

In this study, the molecular analysis was based on the amplification and sequencing of a variable region of the mitochondrial gene, which identified the species as belonging to the *Acropora tenuis* group. However, as it has been stated previously, phylogenetic analysis based solely on the mitochondrial gene is insufficient for proper species assignment (Huang et al. 2008; Quek et al. 2024; Ramírez‐Portilla et al. 2022). A sequence of *A*. *yongei* was also grouped close to the specimen's sequence, in agreement with previous observations of the relatedness of this species with *A. tenuis* (Bridge et al. 2024). This observation is also in agreement with the limitation of the mitochondrial region to accurately assess the species in the genus *Acropora*, and stresses the preliminary nature of the molecular assignment of the specimen in the *tenuis's* group. However, the morphological similarities of the non‐native *Acropora* with *A. tenuis*, and its marked differences with *A. yongei* , suggest the identification in the *tenuis*'s group.

There is some historical confusion in the identification of the species *Acropora tenuis* . Bridge et al. (2024) point out that there are numerous recent studies, carried out in geographically distant locations in the Pacific, that refer to the species *A. tenuis* , and that it is quite likely that many of these do not actually correspond to that species. Several morphological descriptions of *A. tenuis* found in the literature differ greatly from the description of the holotype of Dana (1846). According to the results of Bridge et al. (2024), *A. tenuis* has a fairly restricted geographical distribution in the Pacific, so it is quite likely that many of the species cataloged as *A. tenuis* correspond to other species.

The *Acropora* genus includes some of the fastest growing coral species (Colin and Arneson 1995). *For A. tenuis*, growth rates up to 30 cm in diameter within 3–5 years have been documented (Omori et al. 2008; Iwao et al. 2010). This rapid growth, coupled with a high rate of asexual reproduction via fragmentation, early sexual maturity (4–5 years), and high thermal tolerance in juveniles (Yorifuji et al. 2017), underscores its threat as an invader. Most colonies found in this study had likely reached reproductive maturity (Iwao et al. 2010). Considering all these factors, the presence of colonies from the *Acropora tenuis* group represents a significant threat to the native hard corals and other benthic organisms and consequently, to the biodiversity, trophic structure, and dynamics of the coral reefs of this MPA and potentially to the wider Caribbean. Although all detected colonies were removed from Pescadores reef, it remains the possibility that this species persists in other reefs or hard bottoms within the MNP.

The global aquarium trade has acted as a significant vector for marine introductions for decades, and its footprint is evident in Venezuela. The presence of this exotic coral in the MNP appears directly linked to aquarium‐related activities. *Acropora* and *Euphyllia* are among the most prevalent coral genera in the trade (Sosnowski et al. 2020) and represent some of the highest‐value exports from Indonesia (Akmal et al. 2020), the world's main exporter of live corals. For instance, Indonesian *Acropora* species comprised 19% of all live coral imports to the United States between 2007 and 2016 (Pavitt et al. 2021).

Although Venezuela is a signatory to the Convention on International Trade in Endangered Species of Wild Fauna and Flora (CITES), national biosecurity protocols and the enforcement of import regulations for marine ornamentals remain insufficient. Furthermore, there is limited public awareness of the catastrophic ecological risks associated with the introduction (intentional or accidental) of a non‐native species. Once established, exotic species can displace native taxa and alter food webs and community structures (Wallentinus and Nyberg 2007; David et al. 2017). Consequently, the introduction of exotic species represents one of the major global threats to biodiversity and has a well‐documented socioeconomic impact (Katsanevakis et al. 2018; Essl et al. 2020; Quintana et al. 2023).

The most alarming precedent in Venezuela is the invasion of the soft coral *Unomia stolonifera* (Octocorallia, Alcyonacea), first detected on the eastern coast of the country during the early 2000s (Ruiz Allais et al. 2014, 2021; Benayahu et al. 2021). As in the present research, only a single colony of *U. stolonifera* was initially observed. Currently, *U. stolonifera* has invaded vast areas of shallow and deep bottoms (> 40 m deep), in northeastern Venezuela (Mochima National Park), with further expansions reported in Aragua State. We also found it in the Cuare Wildlife Refuge in Falcón State. The rapid proliferation of the non‐native *Acropora* observed in this study suggests it could follow a similar invasive trajectory if left unmanaged.

**FIGURE A6:**
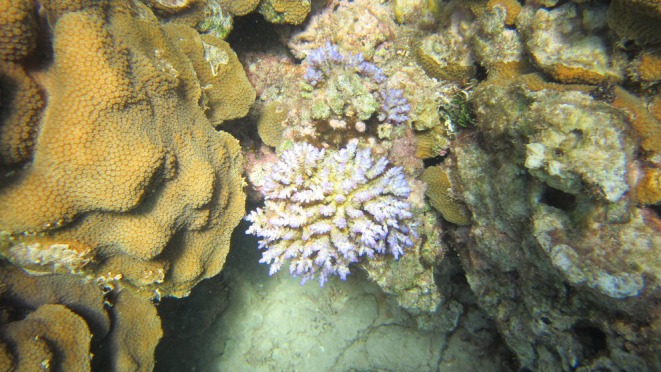
New colony of non‐native *Acropora* at Pescadores Reef (September 2023).

Given the catastrophic ecological damage documented for *U. stolonifera* , all detected colonies of this non‐native A*cropora* were manually removed from the small invaded area, in April 2023. This intervention constitutes a proactive Early detection and Rapid Response (EDRR) effort aimed at containment before secondary dispersal. However, the subsequent emergence of new colonies (Appendix 6: Figure A6) underscores the necessity of a high‐frequency (triannual) monitoring program. Furthermore, it is imperative to expand survey efforts to adjacent cays and across broader bathymetric and geographic ranges within the MPA.

This non‐native *Acropora* is a high‐risk potentially invasive species whose presence in other Caribbean reefs cannot be discounted. In this study, we implemented the foundational pillars of invasive species management: prevention (mitigation spread post‐detection), rapid response (physical removal), and continued surveillance of the affected sites.

The non‐native A*cropora* reported here represents potentially the first exotic hermatypic coral with invasive traits in the Caribbean Sea. A notable historical precedent involves *Lobactis scutaria* (Fungiidae), which was intentionally introduced for research purposes to Discovery Bay, Jamaica, in 1966. Despite numerous eradications attempts between 1970 and 1980, new colonies were detected as late as 2003, highlighting the long‐term persistence of such introductions before final eradication from the Caribbean was achieved (Salimi et al. 2021). Another case includes the invasive zooxanthellate coral *Oculina patagonica* (Oculinidae), first recorded in the Gulf of Mexico (Great Caribbean) in 2015, and widely distributed across the Mediterranean and the Canary Islands (Serrano et al. 2023). Furthermore, non‐reef building corals such as *Tubastrea*, native to the Pacific, have rapidly expanded through the Caribbean since their introduction decades ago (Fenner and Banks 2004; Creed et al. 2016; Hoeksema et al. 2019).

The high architectural complexity and three‐dimensional pattern of the non‐native *Acropora* found in MNP National Park may explain the colonization by a diverse assemblage of associated macroinvertebrates. Such structural frameworks provide critical habitat and refugia that promote higher epifaunal diversity compared to simpler substrates (Grajal and Laughlin 1984, Hinrichsen 1997; Vytopil and Willis 2001; Knowlton et al. 2010). While this association might suggest that the species provides certain ecological functions in its new habitat, these “benefits” are outweighed by the well‐documented risks that established exotic species pose to native community equilibrium. Consequently, the decision to eradicate these colonies from the shallow bottoms of MNP National Park ‐a primary Marine Protected Area in Venezuela‐ was a necessary measure to prioritize the integrity of autochthonous reef frameworks.

Sustained surveillance at both local and regional scales is vital for the early detection and rapid eradication of non‐native species. Furthermore, it is imperative that national authorities enforce more stringent regulatory frameworks and oversight of the aquarium trade. These proactive measures are essential to curb further introductions, mitigate ongoing ecological impacts, and safeguard the long‐term resilience of marine biodiversity in Venezuela and the wider Caribbean.

## Author Contributions

**Estrella Y. Villamizar G:** conceptualization (lead), data curation (lead), funding acquisition (lead), investigation (lead), methodology (equal), project administration (lead), supervision (lead), validation (equal), writing – original draft (lead), writing – review and editing (lead). **Rossana C. Jaspe:** investigation (equal), methodology (equal), writing – original draft (equal), writing – review and editing (equal). **Anaurora Yranzo‐Duque:** conceptualization (equal), investigation (equal), validation (equal), visualization (equal), writing – original draft (equal), writing – review and editing (lead). **Jeannette Pérez‐Benítez:** investigation (equal), methodology (equal), validation (equal), writing – original draft (equal), writing – review and editing (equal). **Yoneira F. Sulbarán:** investigation (equal), methodology (equal). **Samuel Narciso:** investigation (equal), resources (supporting), writing – original draft (equal), writing – review and editing (equal). **Bryan A. Rujano‐Espinoza:** investigation (supporting), writing – original draft (equal). **Carlos Pereira:** investigation (equal), writing – original draft (equal). **María C. Goite:** investigation (equal), writing – original draft (equal). **Flor H. Pujol:** conceptualization (equal), data curation (lead), investigation (equal), methodology (lead), resources (equal), supervision (equal), validation (equal), visualization (equal), writing – original draft (equal).

## Conflicts of Interest

The authors declare no conflicts of interest.

## Data Availability

All relevant data are within the article in the Appendix 7: Tables A2, A3, A4. The catalog number assigned by the Marine Museum of Margarita (Venezuela), to the deposited colony of Acropora tenuis group: MMM‐CNI‐0078, and The number assigned by GenBank: PZ027464, PZ025730.

## Appendix 5

**TABLE A1:** Comparison of morphological diagnostic characters between the non‐native *Acropora* specimens from this study and the original description of *Acropora tenuis* (Dana 1846).

Coral species	Colony shape	Branching	Coenosteum	Axial corallite	Radial corallite	Taxonomic reference
*Acropora tenuis* Dana (1846)	Cespitose, rounded. Sparingly spreading	Branches very slender, 3 in. long, 2 lines thick, subterete and proliferous,	Exterior neatly striate and finely scabrous	Small, a little prominent quite thin	Appressed‐tubiform, irregular, elongate (1½ lines) and slender	Dana (1846) (Original description)
Non‐native Acropora (this study)	Cespito‐Corymbose, rounded	Branches elongate (2.22–5.04″) and thin (0.19–1.98″)	Costate with simple spinules in corallite walls, and between radial corallites	Tubular with rounded margins	Shape can be variable, but mostly cochleariform, with slight differences in length and shape (appressed tubular) from the tip to the base of the branches	This study

## Appendix 7

**TABLE A2:** Percent cover of non‐native *Acropora*, and associated benthic categories within the invaded area at Pescadores reef, Venezuela.

Quadrat number	Healthy non‐native *Acropora* (%)	Unhealthy (or dead) non‐native *Acropora* (%)	Coral rubble and sand	Sand	*Millepora alcicornis*
C1	7	0	0	93	0
C2	33	58	0	9	0
C3	32	68	0	0	0
C4	2	98	0	0	0
C5	6	94	0	0	0
C6	19	78	0	3	0
C7	14	14	72	0	0
C8	10	70	0	11	9
C9	19	79	0	0	2
C10	6	94	0	0	0
C11	3	97	0	0	0
Average	13.7	68.2	6.5	10.5	1.0
Std. Error	3.29	9.97	6.54	8.32	0.82

**TABLE A3:** Morphometric traits of non‐native *Acropora*: branch dimensions (length and diameter) and corallite architecture (external and internal diameters of axial and radial corallites). NM, Not measured.

Main branch	Branches length (cm)	Branches Ø (cm)	Axial ext. Ø (mm)	Axial int. Ø (mm)	Radial ext. Ø (mm)	Radial int. Ø (mm)
1	11.59	0.83	2.10	1.30	NM	NM
2	10.38	0.74	2.30	0.80	NM	NM
3	9.73	0.86	2.80	2.30	NM	NM
4	7.00	0.48	2.10	0.80	NM	NM
5	9.75	0.73	2.20	1.30	NM	NM
6	8.22	1.00	2.26	1.20	NM	NM
7	11.15	1.15	2.10	0.90	NM	NM
8	11.86	1.05	2.20	1.00	NM	NM
9	8.40	0.58	1.20	1.00	NM	NM
10	11.86	1.05	2.70	1.10	NM	NM
11	9.31	0.61	2.20	1.00	2.1	1.2
12	9.25	0.68	2.70	1.20	1.3	0.5
13	7.92	0.48	2.30	0.90	1.7	0.9
14	8.47	0.65	2.20	0.70	1.5	0.6
15	11.01	0.47	4.50	2.10	2	1.4
16	12.81	0.66	2.60	1.30	1.4	0.6
17	6.78	0.72	2.70	1.00	2	0.9
18	8.40	0.49	1.80	1.00	1.6	1.2
19	5.64	0.49	2.40	0.60	1.9	0.8
Average	9.45	0.72	2.39	1.13	1.68	0.86
Std. error	0.45	0.05	0.14	0.10	0.27	0.31
Max	12.81	1.15	4.50	2.30	2	1.4
Min	5.64	0.47	1.20	0.60	1.3	0.5

**TABLE A4:** Results of size estimation methods for non‐native *Acropora* colonies: (a) in situ measurements and (b) photographic analysis.

**A**. Size of non‐native *Acropora* colonies (*n* = 11) measured in situ			
At Pescadores Key—Morrocoy National Park, Venezuela—April, 2023			
Colony number	Cross‐sectional max. diameter (cm)	Transv. diameter (cm)	Height (cm)
1	20	15	10
2	5	5	5
3	15	10	10
4	15	10	15
5	20	20	15
6	20	20	15
7	35	25	20
8	20	15	25
9	20	15	25
10	20	15	25
11	15	15	15
Average	18.64	15.00	16.36
Std. deviation	2.14	1.65	2.03
Maximum	35.00	25.00	25.00
Minimum	5.00	5.00	5.00

**B**. Size of non‐native *Acropora* colonies measured via photography analysis. Pescadores Key—Morrocoy National Park, Venezuela	
Colony number	Cross‐sectional max. diameter (cm)
1	23.57
2	22.10
3	23.57
4	8.10
5	8.91
6	8.64
7	16.08
8	9.20
9	6.80
10	23.00
11	25.88
12	19.41
13	27.00
14	19.20
15	10.00
Average	16.76
Std. deviation	7.40
Maximum	27.00
Minimum	6.80
